# Supplementary material for: Analysis of PPARγ Signaling Activity in Psoriasis
Source: Int J Mol Sci. 2021 Aug 10;22(16):8603. doi: 10.3390/ijms22168603 (PMC8395241; doi:10.3390/ijms22168603)
Supplement: Supplementary file 1 [file ijms-22-08603-s001.zip › Supplemental materials_Analysis of PPARg signaling activity in psoriasis/Pathway models/Models images and html files/Differentiation of psoriatic T cells/104089.html]

IL23R


# Protein IL23R

|  |  |
| --- | --- |
| URN | urn:agi-llid:149233 |
| Total Entities | 0 |
| Connectivity | 790 |
| Name | IL23R |
| Description | interleukin 23 receptor |
| Notes | The protein encoded by this gene is a subunit of the receptor for IL23A/IL23. This protein pairs with the receptor molecule IL12RB1/IL12Rbeta1, and both are required for IL23A signaling. This protein associates constitutively with Janus kinase 2 (JAK2), and also binds to transcription activator STAT3 in a ligand-dependent manner. [provided by RefSeq, Jul 2008] |
| Primary Cell Localization | Plasma membrane |
| Class | Receptor |

---

|  |  |
| --- | --- |
| Pathway | Th17-Cell Differentiation |
|  | AHR in Intestinal Cell Antimicrobial Barrier Maintenance |
|  | T-lymphoid precursor cell -> CD8+ Naive T-cell Surface Expression Markers |
|  | Lymphocyte Mediated Myocardial Injury in Myocarditis |
|  | Proteins Involved in Psoriasis |
|  | Genes with Mutations Associated with Psoriasis |
|  | T-Cells Differentiation Block in Psoriasis |
|  | Th17-Cell Function in Systemic Lupus Erythematosus |
|  | Th17-Cell Activation in Crohn's Disease |
|  | Proteins Involved in Inflammatory Bowel Diseases |
|  | Genes with Susceptibility for Crohn's Disease and Ulcerative Colitis |
|  | Il17 Signaling Related Neutrophilia in Asthma |
|  | Th17-Cell Differentiation in Asthma |
|  | Th17-Cell and Th1 Immune Responsein Psoriatic Arthritis |
|  | Proteins Involved in Psoriatic Arthritis |
|  | Th17-Cell Numbers Reduction in HIV |
|  | Genes with Mutations Associated with Psoriatic Arthritis |
|  | IL23A and IL17A Provoke Cancer-Associated Inflammation |
|  | STAT3 Facilitates the Function of Treg Cells and Cancer Progression |
|  | IL23R -> STAT3/NF-kB Signaling |
|  | PPAR Psoriasis |
|  | pprarg neg, uknown targets, ps-positive |
|  | prarg neg,ukn expres targets, ps-positive |
|  | PPARG negative targets |
|  | pparg expr target\_regulators |
|  | Neighbors of tumor progression |
|  | Neighbors of disease severity |
|  | Neighbors of cancer incidence |
|  | Neighbors of gastric cancer risk |
|  | Neighbors of tumor progression |
|  | Neighbors of disease severity |
|  | Neighbors of cancer incidence |
|  | Neighbors of gastric cancer risk |
|  | Neighbors of cell development |
|  | Neighbors of immune response |
|  | Neighbors of cell population |
|  | Neighbors of T-cell development |
|  | Neighbors of inflammatory response |
|  | Neighbors of cell function |
|  | Neighbors of adaptive immune response |
|  | Neighbors of cell survival |
|  | Neighbors of cell phenotype |
|  | Neighbors of T-cell proliferation |
|  | Neighbors of T-cell activation |
|  | Neighbors of tumor growth |
|  | Neighbors of cell count |
|  | Neighbors of cell infiltration |
|  | Neighbors of bone remodeling |
|  | Neighbors of ossification |
|  | Neighbors of T-cell function |
|  | Neighbors of cell transdifferentiation |
|  | Neighbors of cell growth |
|  | Neighbors of hemato-encephalic barrier |
|  | Neighbors of cellular immune response |
|  | Neighbors of cell differentiation |
|  | Neighbors of psoriasis |
|  | Neighbors of inflammatory disease |
|  | Neighbors of rheumatoid arthritis |
|  | Neighbors of fibrosis |
|  | Neighbors of colitis |
|  | Neighbors of arthritis |
|  | Neighbors of inflammatory bowel disease |
|  | Neighbors of injury |
|  | Neighbors of carcinogenesis |
|  | Neighbors of multiple sclerosis |
|  | Neighbors of inflammation |
|  | Neighbors of hyperplasia |
|  | Neighbors of myocardial infarction |
|  | Neighbors of reperfusion injury |
|  | Neighbors of airway inflammation |
|  | Neighbors of hepatitis |
|  | Neighbors of infection |
|  | Neighbors of preeclampsia |
|  | Neighbors of encephalomyelitis |
|  | Neighbors of autoimmune disease |
|  | Neighbors of hepatocellular carcinoma |
|  | Neighbors of autoimmunity |
|  | Neighbors of gastric cancer |
|  | Neighbors of experimental autoimmune encephalomyelitis |
|  | Neighbors of systemic lupus erythematosus |
|  | Neighbors of collagen-induced arthritis |
|  | Neighbors of kidney disease |
|  | Neighbors of stroke |
|  | Neighbors of cancer |
|  | Neighbors of neoplasm |
|  | Neighbors of coronary artery disease |
|  | Neighbors of rupture |
|  | Neighbors of ovarian cancer |
|  | Neighbors of lung |
|  | Neighbors of skin |
|  | Neighbors of intestine |
|  | Neighbors of colon |
|  | Neighbors of breast |
|  | Neighbors of stomach |
|  | Neighbors of ileum |
|  | Neighbors of oxidized LDL |
|  | Neighbors of IL1 family |
|  | Neighbors of transforming growth factor |
|  | Neighbors of TLR |
|  | Neighbors of cytokine |
|  | Neighbors of interferon |
|  | Neighbors of JAK |
|  | Neighbors of LXR |
|  | Neighbors of inflammatory cytokine |
|  | Neighbors of IL23 |
|  | Neighbors of T-cell receptor |
|  | Neighbors of IL1R |
|  | Neighbors of IL12 |
|  | Neighbors of Notch |
|  | Neighbors of NOS |
|  | Neighbors of RA receptor |
|  | Overview. Diseases of the skin (chapter 11) |
|  | Overview. Diseases of the digestive system (chapter 10) |
|  | 1\_Differentiation of psoriatic T cells |
|  | 4\_1\_Polymorphisms associated with inflammatory bowel diseases |
|  | Differentiation of psoriatic T cells |

---

|  |  |
| --- | --- |
| Group | Genes with Mutations Associated with Psoriasis |
|  | Genes Associated with Systemic Lupus Erythematosus |
|  | Genes with Mutations Associated with Graves Ophthalmopathy |
|  | Genes with Mutations Associated with Inflammatory Bowel Diseases |
|  | Genes Associated with Ulcerative Colitis |
|  | Genes with Mutations Associated with Psoriatic Arthritis |

---

|  |  |
| --- | --- |
| MedScan ID | 149233 |
|  | 654295 |

---

|  |  |
| --- | --- |
| LocusLink ID | 149233 |
|  | 209590 |
|  | 689932 |
|  | 100364993 |

---

|  |  |
| --- | --- |
| Alias | interleukin-23 receptor |
|  | IL-23R |
|  | OTTHUMP00000010660 |
|  | OTTMUSP00000038453 |
|  | LOC100364993 |
|  | interleukin IL23R |
|  | LOC683816 |
|  | IL23 receptor |
|  | interleukin-23R |
|  | LOC100912239 |
|  | interleukin IL23 receptor |
|  | IL-23 receptor |
|  | interleukin 23 receptor-like |
|  | interleukin 23 receptor; interleukin-23 receptor |
|  | interleukin 23 receptor |
|  | IL23R |

---

|  |  |
| --- | --- |
| GO ID | 0019955 |
|  | 0004896 |
|  | 0005143 |
|  | 0042019 |
|  | 0042020 |
|  | 0019221 |
|  | 0050829 |
|  | 0006954 |
|  | 0038155 |
|  | 0032693 |
|  | 0051135 |
|  | 0001916 |
|  | 0042102 |
|  | 0002827 |
|  | 2000330 |
|  | 2000318 |
|  | 0042104 |
|  | 0010536 |
|  | 0002230 |
|  | 0032725 |
|  | 0032729 |
|  | 0032735 |
|  | 0032740 |
|  | 0043382 |
|  | 0032819 |
|  | 0045672 |
|  | 0042531 |
|  | 0042509 |
|  | 0034341 |
|  | 0032496 |
|  | 0009897 |
|  | 0072536 |
|  | 0005886 |
|  | 0043235 |
|  | 0007259 |
|  | 0002376 |
|  | 0045087 |
|  | 0002443 |
|  | 0016021 |
|  | 0005887 |
|  | 0016020 |
|  | 0010535 |
|  | 0042517 |
|  | 0042520 |
|  | 0042523 |
|  | 0042510 |
|  | 0004872 |
|  | 0007165 |
|  | 0005622 |

---

|  |  |
| --- | --- |
| KEGG ID | hsa:149233 |
|  | mmu:209590 |
|  | rno:689932 |

---

|  |  |
| --- | --- |
| Organism | Homo sapiens {Organism urn:agi-taxid:9606} |
|  | Mus musculus {Organism urn:agi-taxid:10090} |
|  | Rattus norvegicus {Organism urn:agi-taxid:10116} |
|  | Mus musculus |
|  | Homo sapiens |
|  | Rattus norvegicus |

---

|  |  |
| --- | --- |
| Mouse chromosome position | 6 |

---

|  |  |
| --- | --- |
| OMIM ID | 607562 |
|  | 612261 |
|  | 605606 |
|  | 177900 |
|  | 266600 |

---

|  |  |
| --- | --- |
| Rat chromosome position | 4q31 |

---

|  |  |
| --- | --- |
| Hugo ID | 19100 |
|  | HGNC:19100 |

---

|  |  |
| --- | --- |
| Human chromosome position | 1p31.3 |

---

|  |  |
| --- | --- |
| Swiss-Prot Accession | Q5VWK5 |
|  | Q5VWK5.3 |
|  | G5E8U4 |
|  | Q8K4B4 |
|  | Q8K4B4.1 |
|  | C9JGX4 |
|  | Q4VGP1 |
|  | Q4VGP2 |
|  | Q4VGP3 |
|  | Q4VGP4 |
|  | Q4VGP5 |
|  | Q4VGP6 |
|  | Q5VWK7 |
|  | Q8IW84 |
|  | Q8NFQ9 |
|  | Q96AS1 |

---

|  |  |
| --- | --- |
| GenBank ID | NC\_000001 |
|  | XR\_001736993 |
|  | XM\_011540790 |
|  | XP\_011539092 |
|  | XM\_011540791 |
|  | XP\_011539093 |
|  | NM\_144701 |
|  | NP\_653302 |
|  | XM\_005270516 |
|  | XP\_005270573 |
|  | NG\_011498 |
|  | AB542702 |
|  | BAJ13471 |
|  | AL109843 |
|  | AL389925 |
|  | CH471059 |
|  | EAX06501 |
|  | EAX06502 |
|  | AF461422 |
|  | AAM44229 |
|  | AM990313 |
|  | CAQ51232 |
|  | AM990314 |
|  | CAQ51233 |
|  | AM990315 |
|  | CAQ51234 |
|  | AM990316 |
|  | CAQ51235 |
|  | AM990317 |
|  | CAQ51236 |
|  | AM990318 |
|  | CAQ51237 |
|  | AM990319 |
|  | CAQ51238 |
|  | AM990320 |
|  | CAQ51239 |
|  | AM990321 |
|  | CAQ51240 |
|  | AM990322 |
|  | CAQ51241 |
|  | AM990323 |
|  | CAQ51242 |
|  | AM990324 |
|  | CAQ51243 |
|  | AM990325 |
|  | CAQ51244 |
|  | AM990326 |
|  | CAQ51245 |
|  | AM990327 |
|  | CAQ51246 |
|  | AM990328 |
|  | CAQ51247 |
|  | AM990329 |
|  | CAQ51248 |
|  | AM990330 |
|  | CAQ51249 |
|  | AM990331 |
|  | CAQ51250 |
|  | AM990332 |
|  | CAQ51251 |
|  | AM990333 |
|  | CAQ51252 |
|  | AM990334 |
|  | CAQ51253 |
|  | AM990335 |
|  | CAQ51254 |
|  | AM990336 |
|  | CAQ51255 |
|  | AM990337 |
|  | CAQ51256 |
|  | AY937250 |
|  | AAY18345 |
|  | AY937251 |
|  | AAY18346 |
|  | AY937252 |
|  | AAY18347 |
|  | AY937253 |
|  | AAY18348 |
|  | AY937254 |
|  | AAY18349 |
|  | AY937255 |
|  | AAY18350 |
|  | BC016829 |
|  | AAH16829 |
|  | BC040720 |
|  | AAH40720 |
|  | BC142955 |
|  | FM212237 |
|  | CAR79024 |
|  | FM212238 |
|  | CAR79025 |
|  | FM212239 |
|  | CAR79026 |
|  | FM212240 |
|  | CAR79027 |
|  | FM212241 |
|  | CAR79028 |
|  | Q5VWK5 |
|  | NC\_000072 |
|  | NM\_144548 |
|  | NP\_653131 |
|  | AC124758 |
|  | AC159129 |
|  | CH466523 |
|  | EDK98806 |
|  | AF461423 |
|  | AAM44230 |
|  | BC112425 |
|  | AAI12426 |
|  | Q8K4B4 |
|  | NC\_005103 |
|  | XM\_001072576 |
|  | XP\_001072576 |
|  | AC\_000072 |
|  | XM\_003753910 |
|  | XP\_003753958 |
|  | AABR07060866 |
|  | AABR07060867 |
|  | AABR07060868 |
|  | AAHX01030717 |
|  | AAHX01030718 |
|  | XM\_011540789 |
|  | XP\_011539091 |
|  | NC\_018912 |
|  | AMYH02001199 |
|  | AMYH02001200 |
|  | BC166698 |
|  | AAI66698 |
|  | AC\_000028 |
|  | AAHY01053986 |
|  | AAHY01053987 |
|  | AAHY01053988 |
|  | AAHY01053989 |
|  | AAHY01053990 |
|  | AC\_000133 |
|  | CAI22678 |
|  | CAI22679 |
|  | CAH70406 |
|  | CAH70408 |
|  | AABR06031415 |
|  | AABR06031416 |
|  | NT\_032977 |
|  | NW\_001838579 |
|  | NW\_921351 |
|  | AC\_000044 |
|  | NT\_039353 |
|  | NW\_001030811 |
|  | NW\_047693 |
|  | AABR03032103 |
|  | AABR03032115 |
|  | AABR03032433 |
|  | AABR03032994 |
|  | AABR03033128 |
|  | AABR03033382 |
|  | AABR03033708 |
|  | AABR03033724 |
|  | AABR03033734 |
|  | AABR03033826 |
|  | AABR03033883 |
|  | AABR03034599 |
|  | AABR03034647 |
|  | AABR03034738 |
|  | AABR03034919 |
|  | AABR03035608 |
|  | AABR03035767 |
|  | AABR03036073 |
|  | AABR03036074 |
|  | AABR03036372 |
|  | AABR03036455 |
|  | AABR03036509 |
|  | AABR03037599 |
|  | AABR03037691 |
|  | AABR03037915 |
|  | AABR03039511 |
|  | AABR03039880 |
|  | AABR03039937 |
|  | XM\_005270512 |
|  | XP\_005270569 |
|  | XM\_005270517 |
|  | XP\_005270574 |
|  | ABBA01074519 |
|  | ABBA01074520 |
|  | ABBA01074521 |
|  | ABBA01074522 |

---

|  |  |
| --- | --- |
| Swiss-Prot ID | IL23R\_HUMAN |
|  | IL23R\_MOUSE |

---

|  |  |
| --- | --- |
| Cell Localization | Cell membrane |
|  | Plasma membrane |

---

|  |  |
| --- | --- |
| Ensembl ID | ENSG00000162594 |
|  | ENSP00000321345.5 |
|  | ENST00000347310.9 |
|  | ENSP00000490340.1 |
|  | ENST00000637002.1 |
|  | ENSMUSG00000049093 |
|  | ENSMUSP00000113342.1 |
|  | ENSMUST00000118364.1 |
|  | ENSRNOG00000007544 |
|  | ENSRNOP00000010151.5 |
|  | ENSRNOT00000010151.5 |
|  | ENSP00000321345 |
|  | ENST00000347310 |
|  | ENSP00000387640 |
|  | ENST00000425614 |
|  | ENSMUSP00000113342 |
|  | ENSMUST00000118364 |
|  | ENSRNOP00000010151 |
|  | ENSRNOT00000010151 |

---

|  |  |
| --- | --- |
| MGI ID | MGI:2181693 |
|  | 2181693 |

---

|  |  |
| --- | --- |
| RGD ID | 1586368 |

---

|  |  |
| --- | --- |
| Unigene ID | Mm.221227 |
|  | Hs.677426 |
|  | Rn.134349 |

---

|  |  |
| --- | --- |
| Homologene ID | 16930 |

---

|  |  |
| --- | --- |
| Shape | Stick-vertex |

---

|  |  |
| --- | --- |
| IPI ID | IPI00303045 |
|  | IPI00479853 |
|  | IPI00815647 |
|  | IPI00816048 |
|  | IPI00816154 |
|  | IPI00816175 |
|  | IPI00816507 |
|  | IPI00987402 |
|  | IPI00170196 |
|  | IPI00776919 |
|  | IPI00192508 |

---
